# Assessment of disease activity using a whole-body MRI derived radiological activity index in chronic nonbacterial osteomyelitis

**DOI:** 10.1186/s12969-021-00620-3

**Published:** 2021-08-14

**Authors:** Martina Capponi, Denise Pires Marafon, Flaminia Rivosecchi, Yongdong Zhao, Manuela Pardeo, Virginia Messia, Laura Tanturri de Horatio, Paolo Tomà, Fabrizio De Benedetti, Antonella Insalaco

**Affiliations:** 1 Division of Rheumatology, Ospedale Pediatrico Bambino Gesù, IRCCS (ERN-RITA center), Rome, Italy; 2 Division of Radiology, Ospedale Pediatrico Bambino Gesù, IRCCS (ERN-RITA center), Rome, Italy; 3 Seattle Children’s Hospital, Department of Pediatrics, University of Washington, and Center for Clinical and Translational Research, Seattle Children’s Research Institute, Seattle, WA USA

**Keywords:** Chronic nonbacterial osteomyelitis, Whole body magnetic resonance imaging, CROMRIS, PGA, Disease activity, Children

## Abstract

**Background:**

Based on the recently developed ChRonic nonbacterial Osteomyelitis MRI Scoring tool (CROMRIS), we developed a radiological activity index (RAI-CROMRIS) to obtain a quantification of the overall bone involvement in individual patients.

**Methods:**

Whole Body Magnetic Resonance Imaging (WB-MRI) images were scored according to parameters included in the RAI-CROMRIS: bone marrow hyperintensity, signal extension, soft tissue/periosteal hyperintensity, bony expansion, vertebral collapse. These parameters were evaluated for each bone unit yielding a score from 0 to 7 and summed up as RAI-CROMRIS including all bone units. We assessed clinical disease activity using a physician global assessment (PGA) and radiological findings in 76 treatment-naïve patients; 46 of 76 were evaluated at 6 and 12 months after initial WB-MRI. Quantitative variables were compared using the Mann-Whitney U test for unmatched groups and the Wilcoxon signed-rank test for paired groups. Correlation was evaluated using Spearman’s rank coefficient (r_s_).

**Results:**

There was a significant correlation between RAI-CROMRIS and PGA (*r*_s_ = 0.32; *p* = 0.0055), between RAI-CROMRIS and presence of elevated erythrocyte sedimentation rate (*p* = 0.013) and C-reactive protein (*p* = 0.0001) at baseline. The RAI-CROMRIS decreased from a median of 17 at baseline to 12 at 6 months (*p* = 0.004) and remained stable (median 11) at 12 months. A correlation between the RAI-CROMRIS and the PGA was observed at baseline (*r*_s_ = 0.41; *p* = 0.004) and during follow up at 6 months (*r*_s_ = 0.33; *p* = 0.025) and 12 months (*r*_s_ = 0.38; *p* = 0.010). The baseline RAI-CROMRIS (median 20) was significantly higher in patients who subsequently received bisphosphonates than in patients who received other treatments (median 12) and decreased significantly after bisphosphonates (*p* = 0.008).

**Conclusions:**

The RAI-CROMRIS was correlated with clinical and laboratory measures of disease activity showing significant short-term changes following treatment with bisphosphonates. This tool could be used in clinical practice and clinical trials after validation.

**Supplementary Information:**

The online version contains supplementary material available at 10.1186/s12969-021-00620-3.

## Background

Chronic non-bacterial osteomyelitis (CNO) is an autoinflammatory bone disease, characterized by sterile bone lesions due to an abnormal regulation of the innate immune response resulting in osteoclast differentiation and activation, osteolysis and bone remodeling [1, 2]. It was first described in 1972 as an unusual form of subacute and chronic symmetrical osteomyelitis [3]. Nowadays the term chronic nonbacterial osteomyelitis is preferred [4, 5].

Incidence rate of CNO varies 1–4 per million children from different reports [6] and an incidence of approximately 1:1,000,000 [7] or 2–5% of all osteomyelitis cases [8]. However, this may be underestimated due to low awareness. The average age of onset ranges between 7 and 12 years [4, 9] with a preference for the female gender [9, 10]. The disease is characterized by bone inflammation. The etiopathogenesis still remains unclear. It is believed to be polygenic and multifactorial leading to an imbalanced expression of anti-inflammatory/immunomodulating (IL-10 and IL-19) and proinflammatory (IL-1β, IL-6, TNF-α, IL-20) cytokines [2, 11, 12].

Clinical presentation and severity of CNO vary significantly from asymptomatic subjects at one end to patients with multiple recurrent lesions with significant functional impact at the other end [13, 14]. The cardinal feature is bone pain. Onset is often insidious and patients may or may not experience concomitant systemic symptoms including low-grade fever and generalized malaise [9, 15]. About 30% of patients had unifocal disease at diagnosis but most of them developed new lesions during the follow-up period [16]. Only 25–40% of individuals have symmetrical bone involvement [4]. Finally, a subset of CNO patients exhibit extra-osseous inflammatory organ involvement, including psoriasis and palmoplantar pustulosis (~ 8%), severe acne (~ 10%) and inflammatory bowel disease (~ 10%) [13, 14, 16, 17].

The diagnosis of CNO still remains a diagnosis of exclusion and a bone biopsy is often advocated to confirm the diseases. Imaging is becoming increasingly important in the diagnosis and follow-up of these patients [18, 19]. Inflammatory lesions in CNO may be detected by conventional radiographs with low sensitivity [20]. Computed tomography (CT) may show lytic lesion and sclerosis at a higher sensitivity than radiograph in CNO. Its use is limited due to the high radiation dose. Bone scintigraphy and positron emission tomography (PET)-CT are sometimes used to offer a whole-body level assessment in order to identify lesions simultaneously [21]. However, both are radiation-based techniques. Whole-body magnetic resonance imaging (WB-MRI) represents the current gold standard in the diagnosis and management of CNO patients. It allows the assessment of multiple affected sites simultaneously through typical findings of marrow edema (hypointense on T1-weighted images and hyperintense on T2-weighted images, or short tau inverse recovery [STIR] during the active phase of the disease) [22, 23]. MRI can also demonstrate associated periosteal reaction, soft tissue inflammation and transphyseal disease. In addition, clinically asymptomatic lesions, also known as “silent” lesions, can often be detected by MRI. WB-MRI is radiation-free and well suited for surveillance and monitoring of therapeutic response; however it requires sedation in younger children.

Recently the ChRonic nonbacterial Osteomyelitis MRI Scoring (CROMRIS) was developed as a standardized evaluation tool of WB-MRI by an international group of pediatric radiologists [24]. It includes the most commonly described characteristics seen in children with CNO from MRI, such as signal intensity, size of signal abnormality within bone marrow, and associated features on MRI. Grading of severity of the findings was discussed by 11 pediatric radiologists through monthly conference calls and a consensus meeting and an atlas was created.

Based on this tool, we developed a radiological activity index (RAI-CROMRIS) to obtain a quantitative measure of bone involvement at multiple sites in individual patients with the aim to measure the “entire radiological burden” of the disease at a whole-body level.

## Material and methods

### Patients

We retrospectively analyzed 76 patients with CNO followed at the division of Rheumatology of “Ospedale Pediatrico Bambino Gesù” from April 2012 to September 2019. Inclusion criteria were: fulfilling the CNO criteria proposed by Jansson et al. [25] and WB-MRI performed at baseline, before starting any specific treatment. Complete clinical data were collected as described below.

Clinical disease activity was evaluated using a physician’s global assessment (PGA), based on a 5-point scoring system (inactive, minimal, mild, moderate, severe disease) that included fever, increase of inflammatory markers, presence of pain and/or functional impairment and physician visual analogue scale (VAS) modified from what described in Pardeo et al. [26] (Table 1). The intensity of pain was assessed by the clinician on a VAS ranging from 0 to 10, with 0 as no pain and 10 as maximum pain. Normal ranges used for laboratory data were as follows: C-reactive protein (CRP) < 0.5 mg/dl and erythrocyte sedimentation rate (ESR) ≤15 mm/h. These clinical data were collected for all patients with baseline WB-MRI as well as those who had WB-MRI at follow up.

**Table 1:** Physician’s global assessment (PGA) scoring system

	SCORE	
	0	1
Presence of fever (Temp ≥38 °C)	No	Yes
Presence of pain and/or functional impairment	No	Yes
Increased of inflammatory markers (CRP ≥0.5 mg/dl and/or ESR > 15 mm/h)	No	Yes
Visual analogue scale (VAS) > 5	No	Yes

*CRP* C-reactive protein, *ESR* erythrocyte sedimentation rate

To evaluate clinical disease activity we used a PGA scoring system based on fever, presence of pain and/or functional impairment, increase of inflammatory markers and physician VAS scale. Total score: 0 = inactive, 1 = minimal, 2 = mild, 3 = moderate and 4 = severe

### Whole body MRI protocol and scoring

The WB-MRI protocol is available in Additional Table A_1_. All studies were performed on a 1.5 T AERA MR scanner (Siemens, Erlangen, Germany). A baseline MRI was available for all patients (MRI T0). A WB-MRI was also available at 6 months (MRI T6) and at 12 months (MRI T12) for 46 patients after starting therapy. MRI images were reviewed on a dedicated PACS workstation by two radiologists in consensus reading, with respectively 2 years and 10 years of experience in pediatric radiology. Readers were blinded to patients clinical, history and laboratory data. WB-MRI images were assessed using the CROMRIS as published by Zhao et al. [24] (Additional Table A_2_). The CROMRIS atlas included evaluation of all bone units (the entire skeleton was divided into long bones, complex bones, hand/foot and spine) using 4 different variables: bone marrow hyperintensity, size of signal intensity within each unit/segment graded using relative measurement (small defined as < 25% of estimated volume, medium as 25–50% and large > 50% of estimated volume), hyperintensity of surrounding tissue and bony expansion. Our RAI-CROMRIS include the following parameters for only active CNO lesions: 1) presence of bone marrow hyperintensity on STIR images (score 0–1); 2) presence of signal extension (score 1–3); 3) presence of soft tissue/periosteal hyperintensity (score 0–1); 4) presence of bony expansion (score 0–1); 5) presence of vertebral collapse (score 0–1) and the total score from all active CNO lesions were summed up as the final score on the whole body level. The maximum score for each bone unit is 7 (Table 2). The minimum score for each identified bone unit is 2 as the presence of bone marrow hyperintensity. The RAI-CROMRIS was a practical adaptation of converting a scoring system for individual bones into a quantitative whole-body score for research use. Joint effusion (synovitis), and measures of damage/complications, such as non-CNO bony abnormalities, limb hypertrophy, kyphosis, bony expansion without bone edema and pathological fractures were excluded from the index developed here.

**Table 2:** RAI-CROMRIS

PARAMETERS		SCORE
Hyperintensity within bone marrow	Absent	0
	Present	1
Extension*	< 25%	1
	25–50%	2
	> 50%	3
Soft tissue hyperintensity/periosteal reaction	Absent	0
	Present	1
Bone expansion	Absent	0
	Present	1
Vertebral compression	Absent	0
	Present	1

The parameters included and score assigned to each variable for active CNO lesions. Adding up all parameters a maximum score of seven was obtained for each bone

*In long bones the extension of bone edema < 25% was defined if one part out of five was involved, between 25 and 50% if two parts out of five were involved and of > 50% if three or more parts were involved. In complex bone and on the spine extension was defined as in CROMRIS scoring with 1 point an extension < 25%, 2 points an extension between 25 and 50% and 3 points with an extension > 50%

The RAI-CROMRIS was calculated for all 76 patients who had a WB-MRI at baseline and for all the 46 patients who had WB-MRI at 6 and 12 months. All the patients had complete clinical and laboratory data. An example of whole-body MRI and of the calculation of the RAI-CROMRIS of a patient is shown in Additional Figure A_1_.

### Statistical analysis

Qualitative variables were expressed as absolute frequency and percentage. Proportions were compared by Chi-square test or Fisher’s exact test, as appropriate. Quantitative variables, reported as medians and interquartile ranges (IQR: 1st-3rd quartile), were analyzed using the Mann-Whitney U test for unmatched groups and the Wilcoxon signed-rank test for paired groups.

Correlation between quantitative variables was evaluated using Spearman’s rank coefficient (r_s_) and the strength of the correlation were classified as follows: r_s_   < 0.40 = weak, 0.40–0.59 = moderate, 0.60–0.79 = strong and ≥ 0.80 = very strong [27].

All statistical tests were two-sided; a *p* value < 0.05 was considered as statistically significant. The analyses and graphs were performed using Stata 15.1 software (StataCorp LLC, College Station, Texas USA, 2017).

## Results

### Demographic data, clinical and radiological findings at baseline

The demographic and baseline characteristics of patients are shown in Table 3. We evaluated 76 patients (48 females) with a median age at disease onset of 10.3 years with an overall disease duration of approximately 6 months. At baseline, 75 patients had pain, 33 had functional impairment and 8 had fever. Our patients presented with a median of VAS pain of 5 (IQR 4–6) and had mild disease activity as shown by a median of PGA of 2.0 (IQR 1.5–3.0) on a 5-point scale scoring system. Increased inflammatory markers (ESR and/or CRP) were present in approximately half of the patients.

**Table 3:** Demographic and baseline characteristics of the 76 patients

Demographic characteristics	
Female, n (%)	48 (63.2)
Age at disease onset (yrs), median (IQR)	10.3 (8.4–12.1)
Disease duration at diagnosis (months), median (IQR)	6.3 (2.3–16.9)
Baseline features	
Fever (Temperature ≥ 38 °C), n (%)	8 (10.5)
Pain, n (%)	75 (98.7)
Functional impairment, n (%)	33 (43.4)
VAS, median (IQR)	5 (4–6)
PGA, median (IQR)	2.0 (1.5–3.0)
Extra-osseous involvement, n (%)	
Mucocutaneous (psoriasis, acne, pustolosis)	16 (21.1)
Joints (arthritis, sacroiliitis)	9 (11.8)
Gastrointestinal (IBDs, Irritable Bowel Syndrome)	6 (7.9)
Psychiatric/psychological disorders	10 (13.2)
ESR > 15 mm/h, n (%)	40 (52.6)
CRP ≥0.5 mg/dl, n (%)	41 (54.0)
Number of affected bone units on MRI at T0, median (IQR)	6 (3–9)
RAI-CROMRIS, median (IQR)	14 (8–24)
Vertebral involvement on MRI, n (%)	22 (29.0)
Follow-up at 6 and 12 months	
Number of patients, n (%)	46 (60.5)

*VAS* visual analogue scale, *PGA* physician global assessment, *IBD* intestinal bowel diseasesm, *ESR* erythrocyte sedimentation rate, *CRP* C-reactive protein, *MRI* magnetic resonance imaging

By WB-MRI, the median number of affected bone units was 6 (IQR 3–9) and the median value of the RAI-CROMRIS was 14 (IQR 8–24) (Table 3). The number and distribution of bone lesions in the baseline WB-MRI are shown in Additional Figure A_2_.

### Correlation of the RAI-CROMRIS with clinical and laboratory parameters

Of the 76 patients, 41 were treated with bisphosphonates; among these 28 patients received bisphosphonates monotherapy, 5 patients received concomitant sulfasalazine (SSZ) and/or methotrexate (MTX), 6 patients received biologic agents (tumor-necrosis factor inhibitors, or interleukin-1 inhibitor) and 2 patients received a combination of disease modifying anti rheumatic drugs (DMARDS, SSZ and/or MTX) and biologic agents.

In the group of patients who did not receive bisphosphonates therapy (35/76): 16 patients have been treated with only nonsteroidal anti-inflammatory drugs (NSAIDs), 15 patients received SSZ and/or MTX, 1 patient received biologic agents, 3 patients received SSZ and/or MTX with concomitant biological therapy.

**Fig. 1:**
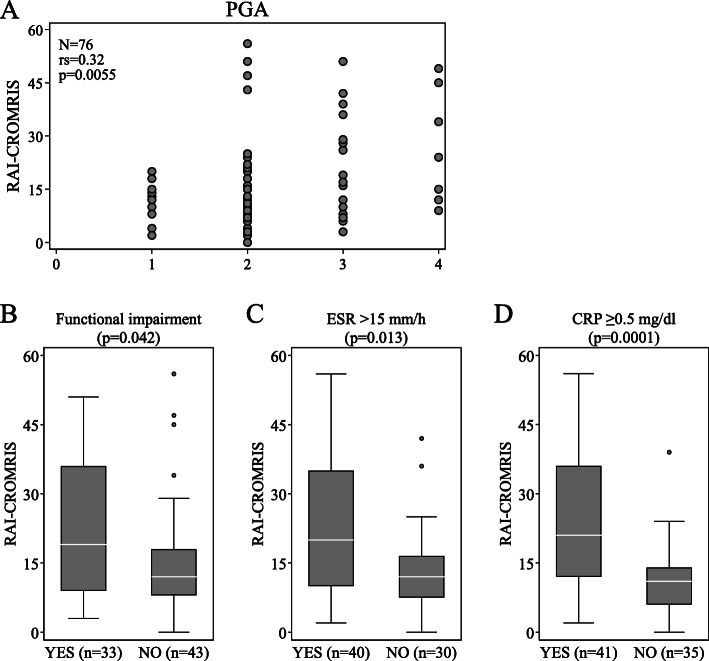
Relationship of the RAI-CROMRIS with clinical and laboratory parameters at baseline. In the 76 patients was analysed the relationship of the RAI-CROMRIS with PGA (**A**), with the presence of functional impairment (**B**), with ESR (**C**) and CRP (**D**). In A the Spearman’s correlation coefficient (*r*_s_) is shown. In B, C and D the *p*-value was calculated with the Mann-Whitney *U* test

At baseline, we found a significant, but weak, correlation between the RAI-CROMRIS and the PGA (*r*_s_ = 0.32; *p* = 0.0055) (Fig. 1A). We also found a significant association of the RAI-CROMRIS with the presence of functional impairment, and with ESR and CRP above the upper limits of normal (Fig. 1B, C and D, respectively). No association of the RAI-CROMRIS was found with presence of fever (temperature ≥ 38 °C) and physician VAS > 5.

Subsequently, we analyzed the clinical and radiological characteristics of the 46 patients followed for 12 months. The 46 subjects were mainly females (29/46, 63%) with a median age at disease onset of 10.2 years (IQR 8.0–12.0) and a median duration of disease at diagnosis of 5.3 months (IQR 2.3–13.1). Disease features are shown in Additional Table A_3__._ The PGA and physician VAS showed a decreasing trend after 6 months from baseline (*p* < 0.0001) and remained substantially stable at 12 months (*p* = 0.65 and *p* = 0.45 versus values at 6 months, respectively) (Additional Table A_3_). CRP was normal at 6 months and at 12 months in the great majority of patients.

**Fig. 2:**
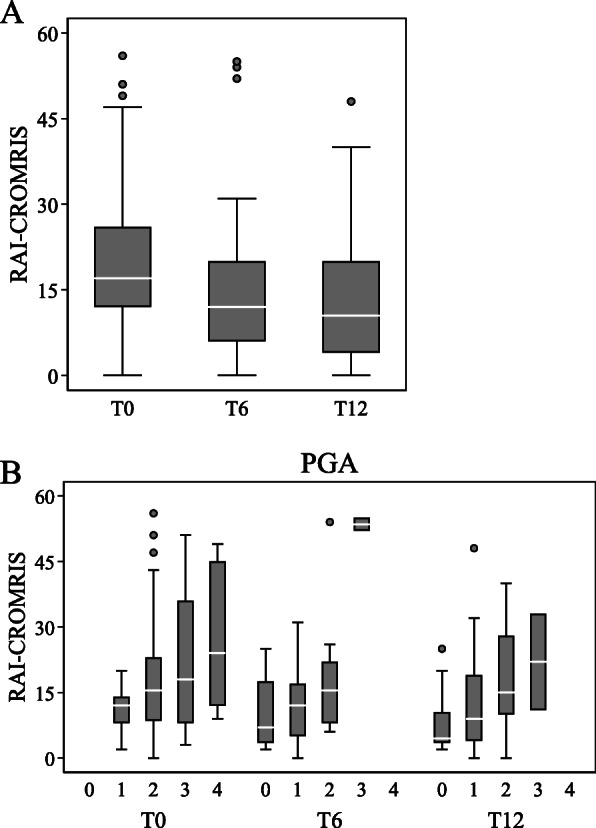
RAI-CROMRIS and relationship with PGA during follow up. The changes in the RAI-CROMRIS during follow-up (**A**) and the relationship with PGA (**B**) of the 46 patients who have MRI at baseline, 6 months and 12 months were evaluated. T0 = baseline, T6 = 6 months, T12 = 12 months

The RAI-CROMRIS decreased from a median of 17 at baseline (IQR 12–26) to a median of 12 at 6 months (IQR 6–20; *p* = 0.004) and remained stable at a median of 11 at 12 months (IQR 4–20; T12 vs baseline *p* = 0.003 and T12 vs T6 *p* = 0.52) (Fig. 2A). The distribution of the PGA scores and the relation with the RAI-CROMRIS at different time points is shown in Fig. 2B. A significant correlation between the RAI-CROMRIS and the PGA was observed at baseline and during follow up with a moderate correlation at baseline (*r*_s_ = 0.41, *p* = 0.004) and a weak correlation at 6 months (*r*_s_ = 0.33, *p* = 0.025) and at 12 months (*r*_s_ = 0.38, *p* = 0.010). We also found that the changes in the RAI-CROMRIS from baseline to 12 months were significantly associated with the changes in the PGA (*r*_s=_ 0.43, *p* = 0.003).

### RAI-CROMRIS at baseline and at 6 months in patients treated with bisphosphonates

As the optimal treatment of CNO has not yet been established, the therapeutic choices for CNO patients are driven by the treating physician’s judgement. Bisphosphonates are usually proposed in current treatment plans for more severe patients [28]. We retrospectively compared RAI-CROMRIS values at baseline and changes at 6 months in patients who received bisphosphonates (*n* = 27) with those who did not (*n* = 19). We found that patients who subsequently received bisphosphonates had higher baseline RAI-CROMRIS compared to patients who received other treatments (median 20, IQR 13–42 vs. median 12, IQR 8–18; *p* = 0.008).

**Fig. 3:**
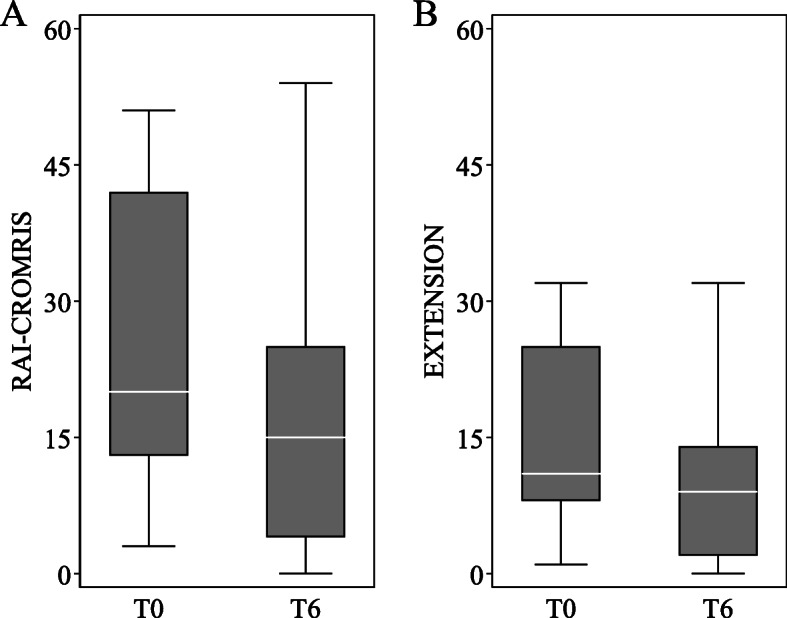
RAI-CROMRIS at baseline and at 6 months in patients treated with bisphosphonates. The distribution of RAI-CROMRIS values (**A**) and of the extension of bone hyperintensity (**B**) at baseline and at 6 months in 27 patients who received bisphosphonates with MRI at baseline (T0) and at 6 months (T6) were analysed

In the patients who received bisphosphonate PGA decreased from a median of 2 (range 1–4) to a median of 1 (range 0–3) and the percentage of patients with abnormal ESR and/or CRP changed from 51.9 to 25.9% and from 59.3 to 11.1%, respectively. A decrease of the RAI-CROMRIS was observed from a median of 20 at baseline (IQR 13–42, range 3–51) to a median of 15 at 6 months (IQR 4–25, range 0–54) (*p* = 0.003 by paired test) (Fig. 3A). The decrease in the score was particularly evident for extension of bone hyperintensity (T0: median = 11, IQR = 8–25, range = 1–32; T6: median = 9, IQR = 2–14, range = 0–32; *p* = 0.0027 by paired test) (Fig. 3B). In addition, the soft tissue hyperintensity observed at baseline in 7 patients, resolved in 5, and persisted in 2 patients at 6 months.

## Discussion

Whole-body MRI (WB-MRI) is one of the mainstays in the diagnosis and management of CNO patients. It is becoming the imaging modality of choice because of its sensitivity in detecting lesions and their characteristic pattern, as well as clinically silent lesions, without ionizing radiation [29]. However, the issue of appropriate interpretation, particularly in children growing bones, has led a number of investigators to propose standardized MRI protocols and scoring systems with the aims to improve reproducibility and inter-examiner variability and, ultimately, diagnosis and staging of the disease [30, 31]. Some of the available scoring systems have shown merits, but also some limitations and they have not been validated. One is an MRI score system for the osteitis lesions used in patients who met the criteria for SAPHO syndrome (Synovitis, Acne, Pustulosis, Hyperostosis, Osteitis syndrome). This score ranges from 0 to 2 with score 0 defined as no bone marrow edema, osteal erosions or synovitis (with or without joint effusion); score 1 defined as the presence of one of these findings; and score 2 defined as the presence of 2 or more findings. In case of more than one osteitis lesion, the lesion with the highest score was used as reference for that patient [30, 32]. In the RINBO index, points are assigned for each of four parameters of interest: the number of radiologic active lesions clustered into 3 categories as unifocal, paucifocal [2–4 lesions] and multifocal [5 or more lesions], the diameter of the largest lesion, the presence of extra medullary involvement, the presence of spinal involvement [33]. Differently from the previous score, the RINBO generates a total score summing the score of the lesions. The main limitation is that the measurement of the size of lesions is not reported as relative to the size of each bone, which is more appropriate, particularly, in children.

We chose to use as a basis of a global quantitative radiological assessment the CROMRIS, a standardized grading of the different features of CNO lesions, irrespective of the bone involved, recently designed by Zhao et al. [24]. It is based on CNO features previously used to score region-specific MRI [31], but with modifications developed, through a consensus process, by 11 pediatric experienced radiologists from 7 different centers and 2 continents (North America and Europe). These authors produced, after a literature review, a complete atlas that consists of detailed definitions and grading of signal intensity, size of signal abnormality within bone marrow and surrounding tissue, physis damage and vertebral compression for each individual bone unit. This method showed excellent reliability and agreement in each category of bones and moderate to substantial reliability and agreement in readings from individual bones [24]. This score was designed to be easily applied in settings with non-experienced radiologists. Therefore, the CROMRIS represents an excellent standardized semi-quantitative scoring system [24]. A total score reflecting the radiological activity of each individual patient has not been proposed. A total score taking all lesions into account is needed because the fluctuating course of CNO implies that new lesions may occur while others are vanishing. Hence, we chose to integrate scores from each bone unit in a comprehensive RAI-CROMRIS that could measure the “entire radiological burden” of the disease at a whole-body level.

Few studies with small cohorts addressed the direct correlation between radiological and clinical findings in CNO patients. A comparison of clinical and radiological findings was previously performed in small cohort without a quantitative radiological score [34, 35]. More recently, the RINBO score was evaluated in 40 patients and found to have a fair agreement with the clinical evaluation of each bone site; however, patients’ medication and follow-up scans were not taken into account [33]. In our study we evaluated the correlation of the RAI-CROMRIS with disease activity in 76 patients before starting treatment and in 46 patients also at two subsequent time points (6 and 12 months). We found a significant association of the RAI-CROMRIS with disease activity parameters including the PGA, presence of functional impairment and increased inflammatory markers in patients at baseline. These observations suggest that the burden of radiological activity, as measured by the RAI-CROMRIS, reflects the overall degree of clinical activity of each individual patient. Examining 46 patients with WB-MRI at baseline and at 6 and 12 months, we found a significant decrease in the RAI-CROMRIS overtime. Importantly, a correlation between the RAI-CROMRIS and the PGA was observed, not only at baseline, but also during follow up at 6 and 12 months, at times when the disease activity was significantly lower, suggesting that this score may be able to reflect minor changes in disease activity.

Several studies have shown the effectiveness of bisphosphonates as a treatment option for patients with significant disease burden, with physical limitations or active vertebral lesions or in those who have persistently active symptoms and abnormal MRI findings who have failed others treatment (NSAID, DMARDS, biologic agents) [28, 31, 36, 37]. This is reflected by the recently developed consensus treatment plans [28]. The patients whom we chose to treat with bisphosphonates, not only had a more severe disease (as shown by more frequent functional impairment and more frequent vertebral involvement), but also had a significantly higher baseline RAI-CROMRIS compared to patients who received other treatments. After 6 months of treatment, the improvement in disease activity was associated with a significant decrease in the RAI-CROMRIS. The decrease in the score was particularly evident for extension of bone hyperintensity, as well as for soft tissue hyperintensity. As hyperintensity is indicative of inflammatory edema, this observation is consistent with the hypothesis of an anti-inflammatory effects of targeting osteoclasts in CNO.

There is no guideline of how frequently whole-body MRI needs to be performed in CNO patients. In the CARRA consensus treatment plan, MRI is strongly recommended to objectively assess disease activity at 6 and 12 months after adjusting therapy [28]. The timing of MRI in this work follows the consensus reached by the CARRA investigators.

There are some limitations of our study. Even if our series is large, the monocentric nature of the study, its observational design, the heterogeneity of the treatments used and the rather long time interval in which the patients were enrolled require caution in drawing definitive conclusions. Although we have used a comprehensive PGA including different variables, such as physician reported outcome, patient reported outcome and increase in acute phase markers, this is not validated. Indeed, there is not yet an internationally validated tool for measuring disease activity in CNO. It should also be noted that bone hyperintensity on STIR sequences may be present in healthy children during growth and, therefore differentiating this physiological hyperintensity from the pathological inflammatory oedema of CNO may be challenging [38]. Bone-marrow-edema like changes in the hand skeleton are reported in healthy children aged 5–16 years in the hand skeleton [39, 40], as well as in feet [41] and pelvis [38]. The low prevalence of bone hyperintensity within these sites in our cohort and the relevant change after treatment argue against over-reading. Further studies are warranted to compare the minimal changes detected in children with CNO with those from healthy children in order to identify the key difference that would be specific to active disease in CNO and to determine the minimum clinically significant difference of MRI findings by using external validation measurements [42].

Furthermore, a potential limitation of the CROMRIS is that the parameters, except extension of hyperintensity in each bone unit, have the same scoring range, which may have diluted an important variable. Also all bone units were considered equally, though hands bones and foot bones were grouped together and assigned the same unbiased weight. Whether these issues are relevant in the evaluation of the total radiological burden of disease and its relation to clinical disease activity remains to be established. Our study provides follow-up information up to 1 year, which is informative on the short term, but longer-term follow-up is needed in a chronic relapsing disease such as CNO. Radiological follow-up studies are lacking. In the only available follow-up study WB-MRI performed more than 10 years after disease onset, revealed radiologically active lesions in more than 50% of patients even in long-term clinical remission [35].

## Conclusions

In conclusion, our results show that the RAI-CROMRIS provides a measure of the overall radiological burden of disease in individual CNO patients and that it is related to clinical and laboratory measures of disease activity, as well as shows significant short-term changes following treatment with bisphosphonates. Larger studies, ideally multicentric, are needed to evaluate the usefulness of the RAI-CROMRIS to stratify patients with CNO based on the severity of the disease, and consequently to guide the most appropriate therapeutic choice, and to evaluate its sensitivity to change following treatments. This radiological score could be included in clinical trials aimed to evaluate the efficacy of targeted treatments in CNO.

## Supplementary Information

**Additional file 1.** Table A1. Sequence parameters of WB-MRI protocol. Table A2. CROMRIS template. Table A3. Clinical disease features during follow up. Fig. A1. Example of whole-body MRI and of the calculation of the RAI-CROMRIS of a patient. Fig. A2. Skeletal involvement.

## Data Availability

The datasets used and analysed during the current study are available from the corresponding author on reasonable request.

## Abbreviations

CNO: Chronic non-bacterial osteomyelitis
CROMRIS: ChRonic nonbacterial Osteomyelitis MRI Scoring tool
CRP: C-reactive protein
DMARDS: Disease modifying anti rheumatic drugs
ESR: Erythrocyte sedimentation rate
MTX: Methotrexate
NSAIDs: Nonsteroidal anti-inflammatory drugs
PET-CT: Positron emission-computed tomography
PGA: Physician global assessment
RAI-CROMRIS: Radiological Activity Index-ChRonic nonbacterial Osteomyelitis MRI Scoring tool
RINBO: Radiological index non-bacterial osteomyelitis
SAPHO syndrome: Synovitis, acne, pustulosis, hyperostosis, osteitis syndrome
STIR: Short tau inverse recovery
SSZ: Sulfasalazine
VAS: Visual analogue scale
WB-MRI: Whole body magnetic resonance imaging
