# Supplementary material for: Assessment of disease activity using a whole-body MRI derived radiological activity index in chronic nonbacterial osteomyelitis
Source: Pediatr Rheumatol Online J. 2021 Aug 14;19:123. doi: 10.1186/s12969-021-00620-3 (PMC8364123; doi:10.1186/s12969-021-00620-3)

**Additional Table A_1_.** Sequence parameters of WB-MRI protocol.

|  | **STIR coronal** | **STIR sagittal** |
| --- | --- | --- |
| TR (ms) | 5000 | 4000 |
| TI (ms) | 160 | 160 |
| TE (ms) | 58 | 58 |
| Resolution (phase x read x slice) (mm^3^) | 1.0x1.0x3.00 | 1.0x1.0x3.00 |
| FOV | 300/500 depending on patient size | 300/500 depending on patient size |
| Base resolution - Matrix | > 256 | > 256 |
| Phase coding | head > feet | anterior > posterior |
| Phase oversampling | 75% | - |
| Flip Angle | 147 | 147 |
| Bandwidth (Hz/px) | 233 | 233 |
| Parallel imaging method (Accel. Factor PE) | GRAPPA (3) | GRAPPA (3) |
| Respiration motion | Free breathing | Free breathing |
| Acquisition time (min) | 2.02 | 2.02 |

**Additional Table A_2_. CROMRIS template.**

Template to assess WB-MRI derived from CROMRIS as published by Zhao Y et al.

| SKELETAL SITES |  |
| --- | --- |
| Complex bones template | Scapula, sacrum (right and left halves), pelvis (divided into ilium, periacetabulum, pubis/ischium), ribs, mandible (right and left halves), skull, patella, clavicle and sterna/manubrium |
| Long bones template | Humerus, radio, ulna, femur, tibia and fibula (each bone divided into proximal epiphysis, proximal metaphysis, diaphysis, distal metaphysis, distal epiphysis) |
| Hand/foot template | Hand considered one unit; foot divided into hindfoot (talus/calcaneus), mid/forefoot (tarsals, metatarsals, phalanges) |
| Spine template | Cervical/thoracic/lumbar vertebral bodies |
| MRI CHARACTERISTICS |  |
| Bone marrow hyperintensity | Absent, < fluid signal, and similar to fluid signal |
| Signal size | Absent, <25%, 25-50%, and >50% of the bone involved |
| Soft tissue or periosteal hyperintensity | Absent or present |
| Bony expansion | Absent or present |
| DAMAGE/COMPLICATION SCORING |  |
| Joint effusion (synovitis) | Absent or present |
| Non-CNO bony abnormalities | Absent or present |
| Limb hypertrophy | Absent or present |
| Vertebral compression | Normal, some height loss, and vertebra plana |
| Kyphosis | Absent or present |
| Bony expansion without bone edema | Absent or present |
| Pathological fracture | Absent or present |
|  |  |
| CONFIDENCE LEVEL | Mark if medium or low |

**Additional Table A_3_.** **Clinical disease features during follow up.**

Characteristics at baseline (T0), after 6 (T6) and 12 months (T12) of the 46 patients who were followed.

|  | **T0** | **T6** | **T12** | **p value*** | | |
| --- | --- | --- | --- | --- | --- | --- |
|  |  |  |  | **T0-T6** | **T0-T12** | **T6-T12** |
| Fever (T ≥38°C), n (%) | 5 (10.9) | 1 (2.2) | 1 (2.2) | 0.20^F^ | 0.20^F^ | 1.0^F^ |
| Pain, n (%) | 45 (97.8) | 37 (80.4) | 30 (65.2) | 0.007^C^ | <0.0001^C^ | 0.10^C^ |
| Functional impairment, n (%) | 21 (45.7) | 5 (10.9) | 6 (13.0) | <0.0001^C^ | 0.001^C^ | 0.75^C^ |
| VAS, median (IQR) | 5 (4-6) | 3 (2-4) | 3 (1-5) | <0.0001 | <0.0001 | 0.45 |
| VAS >5, n (%) | 18 (39.1) | 3 (6.5) | 6 (13.0) | <0.0001^C^ | 0.004^C^ | 0.49^F^ |
| PGA, median (IQR) | 2 (2-3) | 1 (1-2) | 1 (0-2) | <0.0001 | <0.0001 | 0.65 |
| ESR mm/h, median (IQR) | 16 (8-36) | 6 (5-15) | 7 (5-11) | <0.0001 | <0.0001 | 0.94 |
| ESR >15 mm/h, n (%) | 24 (52.2) | 11 (23.9) | 10 (21.7) | 0.005^C^ | 0.002^C^ | 0.80^C^ |
| CRP mg/dl, median (IQR) | 0.59 (0.05-2.05) | 0.04 (0.04-0.11) | 0.05 (0.04-0.13) | <0.0001 | <0.0001 | 0.91 |
| CRP ≥0.5 mg/dl, n (%) | 26 (56.5) | 6 (13.0) | 7 (15.2) | <0.0001^C^ | <0.0001^C^ | 0.77^C^ |
| *p value: Chi-square test (^C^); Fisher’s exact test (^F^). Wilcoxon signed-rank test was used for continuous variables. | | | | | | |

**Additional Figure A_1_. Example of whole-body MRI and of the calculation of the RAI-CROMRIS of a patient.**

**
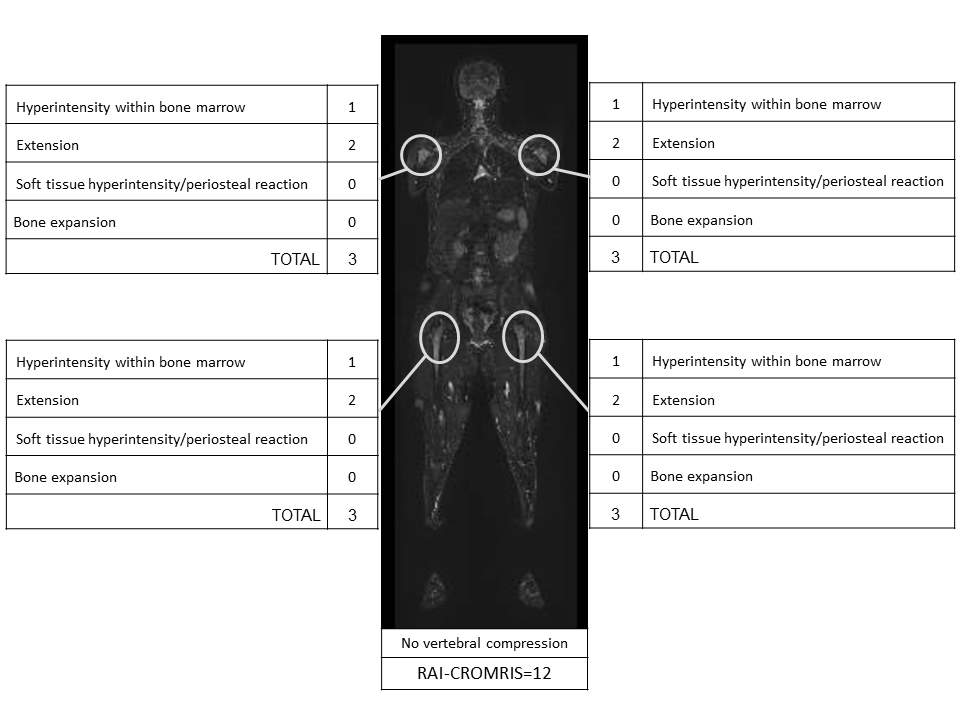
**

**Additional Figure A_2_. Skeletal involvement.**

Number and distribution of bone lesions detected by WB-MRI at baseline.


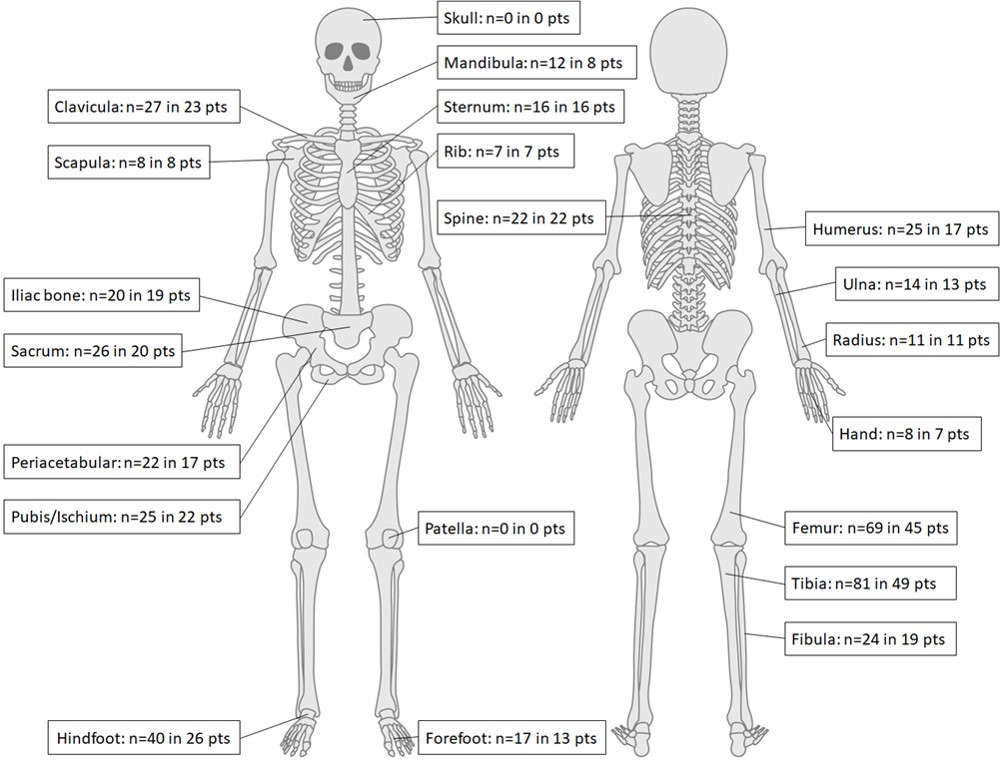

Supplement: Supplementary file 1 — Additional file 1. Table A1. Sequence parameters of WB-MRI protocol. Table A2. CROMRIS template. Table A3. Clinical disease features during follow up. Fig. A1. Example of whole-body MRI and of the calculation of the RAI-CROMRIS of a patient. Fig. A2. Skeletal involvement. [file 12969_2021_620_MOESM1_ESM.docx]
